# Apoptotic cell infusion treats ongoing collagen-induced arthritis, even in the presence of methotrexate, and is synergic with anti-TNF therapy

**DOI:** 10.1186/s13075-016-1084-0

**Published:** 2016-08-11

**Authors:** Francis Bonnefoy, Anna Daoui, Séverine Valmary-Degano, Eric Toussirot, Philippe Saas, Sylvain Perruche

**Affiliations:** 1 INSERM UMR1098, F-25000 Besançon, France; 2 Université de Bourgogne Franche-Comté, SFR FED4234, F-25000 Besançon, France; 3 EFS Bourgogne Franche-Comté, F-25000 Besançon, France; 4 LabEX LipSTIC, ANR-11-LABX-0021, F-25000 Besançon, France; 5 FHU INCREASE, Besançon University Hospital, F-25000 Besançon, France; 6 Pathology Department, Besancon University Hospital, F-25000 Besançon, France; 7 INSERM CIC1431, Clinical Investigation Center Biotherapy, Besançon University Hospital, F-25000 Besançon, France; 8 Rheumatology Department, Besançon University Hospital, F-25000 Besançon, France; 9 UMR1098 INSERM, Etablissement Français du Sang de BFC, 8 Rue du Dr JFX Girod, F-25000 Besançon, France

## Abstract

**Background:**

Apoptotic cell-based therapies have been proposed to treat chronic inflammatory diseases. The aim of this study was to investigate the effect of intravenous (i.v.) apoptotic cell infusion in ongoing collagen-induced arthritis (CIA) and the interaction of this therapy with other treatments used in rheumatoid arthritis (RA), including methotrexate (MTX) or anti-TNF therapy.

**Methods:**

The effects of i.v. apoptotic cell infusion were evaluated in a CIA mouse model in DBA/1 mice immunized with bovine type II collagen. The number and functions of antigen-presenting cells (APC), regulatory CD4^+^ T cells (Treg), and circulating anti-collagen auto-antibodies were analyzed in CIA mice.

**Results:**

Treatment of arthritic mice with i.v. apoptotic cell infusion significantly reduced the arthritis clinical score. This therapeutic approach modified T cell responses against the collagen auto-antigen with selective induction of collagen-specific Treg. In addition, we observed that APC from apoptotic-cell-treated animals were resistant to toll-like receptor ligand activation and favored ex vivo Treg induction, indicating APC reprogramming. Apoptotic cell injection-induced arthritis modulation was dependent on transforming growth factor (TGF)-β, as neutralizing anti-TGF-β antibody prevented the effects of apoptotic cells. Methotrexate did not interfere, while anti-TNF therapy was synergic with apoptotic-cell-based therapy.

**Conclusion:**

Overall, our data demonstrate that apoptotic-cell-based therapy is efficient in treating ongoing CIA, compatible with current RA treatments, and needs to be evaluated in humans in the treatment of RA.

## Background

Rheumatoid arthritis (RA) is an autoimmune disorder characterized by chronic inflammation of the synovial joints leading to the destruction of cartilage, bone, and ligaments [1]. Conventional treatment of RA with disease-modifying anti-rheumatic drugs (DMARD) aims to limit disease symptoms, delay or prevent future joint destruction, and target low disease activity or remission. Low-dose methotrexate (MTX) is the traditional DMARD administered weekly either alone or in combination therapy. MTX has been proven safe and efficient [2]. However, nearly a quarter of patients treated with MTX have to discontinue treatment because of poor responses, adverse effects (e.g., hepatic, gastrointestinal, hematological, renal, or pulmonary toxicity), or both [3, 4]. Biological agents, such as anti-TNF therapy, combined with MTX have significantly improved the treatment of RA. However, again, some RA patients are refractory or contraindicated to these agents [4, 5], and thus, new therapeutic strategies are needed.

Apoptotic cell administration has been shown to control chronic inflammatory disorders by diminishing the pro-inflammatory state and to induce or restore tolerance to auto-antigens by inhibiting pathogenic T or B cell responses and by inducing pro-tolerogenic/regulatory cells [6–8]. Prevention of arthritis by apoptotic cell injection has been reported in mouse and rat models [9–12]. Prevention means that apoptotic cells are infused at the time of arthritic disease induction (i.e., at time of immunization with auto-antigens), which does not mimic the clinical situation. However, intravenous (i.v.) apoptotic cell infusion can be used for experimental treatment of disease, such as in sepsis [13, 14]. These data are interesting, because apoptotic cell administration during the disease (i.e., as treatment) protects mice from sepsis-induced death [13, 14], while infusion 5 days before sepsis (as prevention) worsens mice survival, possibly by decreasing the capacity to secrete interferon (IFN)-γ [15]. As in arthritis models [9–12], sepsis is controlled independently of the apoptotic cell origin [13, 14]. Recently, a phase 1/2a clinical study was conducted in 13 patients who received i.v. donor apoptotic cell infusion the day before allogeneic hematopoietic cell transplantation in order to alleviate the occurrence of acute graft-versus-host disease (GvHD) [16]. The apoptotic cell number infused in patients was transposed from animal models [17]. There was no specific toxicity associated with i.v. apoptotic cell infusion. Historical data on acute GvHD and the available literature suggest promising potential for GvHD prophylaxis [16]. This clinical study opens the way to apoptotic cell-based therapy in other clinical settings already assessed in experimental models, such as RA. Here, we propose to assess whether i.v. apoptotic cell infusion may control ongoing collagen-induced arthritis (CIA) and determine the mechanisms involved by focusing on antigen presenting cells (APC) and regulatory CD4^+^ T cells (Treg).

A major concern with novel therapeutic approaches, such as apoptotic-cell-based therapy, is the interaction with other treatments received simultaneously by the patients. For instance, MTX, the gold standard treatment for RA, may be given alongside biologic agents, including anti-TNF therapy. We have already studied the interactions of i.v. apoptotic cell infusion with immunosuppressive drugs routinely used in the context of allogeneic hematopoietic cell transplantation. Rapamycin (sirolimus) has been shown to exert a synergic effect, while cyclosporine A neutralizes apoptotic-cell-induced allogeneic hematopoietic cell engraftment [18]. This kind of study has to be extended to other conventional drugs in the treatment of RA, such as MTX and anti-TNF agents. We also addressed interactions between i.v. apoptotic cell infusion and MTX or anti-TNF therapy in the CIA model.

## Methods

### Mice

Female DBA/1, (Janvier, Le Genest-Saint-Isle, France) and C57Bl/6 (Charles River Laboratories, L’Arbresle, France) mice, 8–10 week old, were housed in filter-top cages and fed a standard diet with freely available food and sterile water (Plexx, Elst, Netherlands), at the UMR1098 animal facility (agreement number D25-056-7). All experimental studies were approved (number 02831) by the local ethics committee (Comité d’éthique Bisontin en Expérimentation animale, number 58) and the French Ministry of Higher Education and Research (Ministère de l’Enseignement Supérieur et de la Recherche), and was conducted in accordance with the European Union Directive 2010/63.

### Induction of collagen-induced arthritis

The induction of arthritis has been described previously in detail [19]. Briefly, DBA/1 susceptible mice were immunized by subcutaneous injection at the tail base with 100 μL of bovine type II collagen (hereafter called collagen) dissolved in 0.05 M acetic acid (4 mg/mL; MD Bioproducts, Zurich, Switzerland) emulsified in an equal volume of complete Freund’s adjuvant (CFA, 10 mg of *Mycobacterium tuberculosis* (MBT) strain H37Ra (Difco, Detroit, USA) per milliliter of incomplete Freund adjuvant (Sigma Aldrich, St Louis, MO, USA)).

Arthritis developed in all mice 20 to 25 days after collagen immunization. Arthritis severity was determined by daily blinded visual examination of the paws as follows: 0 = no change; 1 = redness or swelling of one toe; 2 = redness or swelling of two toes; 3 = redness or severe swelling of two toes or more digits, erythema, or swelling involving the entire paw; and 4 = redness or swelling of the entire paw, spreading to the ankle. The clinical score for each mouse was the result of the sum of scores for the four limbs (maximum score = 16). When arthritis reached a clinical score of 7–8, mice received apoptotic cells i.v. (5.10e6 cells/300 μL/mouse, or as indicated), or vehicle (300 μL of PBS).

MTX (provided by the University Hospital, Besançon, France), was given intraperitoneally (i.p.) once a week (15 mg/kg), starting on the day of apoptotic cell injection. Anti-TGF-β antibody (clone 1D11 (R&D Systems, Lille, France) or 2G7 clone provided by Prof. L. Chatenoud (Necker Hospital, Paris, France)) was given i.p. on the day of apoptotic cell injection (150 μg/mouse) and 48 h later (100 μg/mouse). Anti-TNF antibody (clone TN3-19.12 (BD Biosciences; Le Pont de Claix, France)) was given i.p. on the day of apoptotic cell injection (300 μg/mouse) and at 72 h, 6 days, and 9 days later (300 μg/mouse/injection). CIA mice that did or did not receive apoptotic cells were killed on days 10 to 12 post treatment and were harvested for blood, lymphoid organs, and ankles for further analysis.

### Induction of apoptotic cells

Cells were issued from the thymus of naïve DBA/1 mice and submitted to a 35-Gy dose of x-ray irradiation (Raycell blood irradiator; Best Theratronic, Ottawa, ON, Canada) followed by 6-h culture in complete DMEM Glutamax-I (Life Technologies, Gaithersburg, MD, USA) supplemented with 10 % heat-inactivated FCS (Life Technologies), 1 % penicillin/streptomycin, 10 mM HEPES buffer (Sigma Aldrich), 10 mM nonessential amino acids (Invitrogen, Cergy Pontoise, France), and 0.05 mM 2-mercaptoethanol (Sigma Aldrich), to allow apoptotic changes to occur before injection [20]. Apoptotic cells were then washed twice in PBS before injection into the tail vein in 300 μL of PBS. Non-treated arthritic mice received PBS as control. Apoptosis was confirmed using fluorescein isothiocyanate (FITC) or phycoerythrin (PE)-conjugated annexin V staining and 7-aminoactinomycin D2 (7-AAD) exclusion (BD Biosciences) and flow cytometry analysis. At the time of injection, apoptotic cells were mainly early-stage apoptotic cells (70–85 % of cells were annexin V^+^ and 7-AAD^–^; less than 10 % of cells were 7-AAD^+^) [20].

### Ex vivo analysis of T cells and antigen presenting cells

Lymphoid organs, spleen, and inguinal and axillary lymph nodes were harvested and dissociated, and erythrocytes were removed by osmotic shock. After washing with PBS, lymphoid cells were directly stained for CD4 (clone RM4-5, BD Biosciences), CD25 (clone PC-61, BD Biosciences) and Foxp3 (clone FJK-16 s, eBioscience) expression following manufacturer’s instructions. Stained cells were analyzed using a FACS Canto II cytometer with Diva software (BD Biosciences).

Cells issued from lymph nodes were also plated and activated using CD3-specific antibodies (Ab) (0.5 μg/mL; clone 145-2C11; Biolegend) or collagen protein as indicated and cultured for 5 days in complete medium. T cell proliferation was then evaluated using 5-Bromo-2’-deoxyuridine (BrdU) incorporation and counting (Perkin Elmer, Waltham, MA, USA). Spleen CD4^+^CD25^+^ T cells were enriched using immuno-magnetic cell sorting (MACS; CD4^+^CD25^+^ Regulatory T Cell Isolation Kit; Miltenyi Biotec, Paris, France) according to manufacturer’s instructions and used in collagen-specific or MBT-specific proliferation assays or in CD3/CD28-stimulated T cell cultures at different concentrations. For collagen-specific or MBT-specific co-cultures, naïve CD4^+^CD25^–^ T cells were isolated from CIA mice by MACS (Miltenyi Biotec) and cultured (100.10e3 cells) with CD11c^+^ dendritic cells (DC) (50.10e3 cells) also isolated from CIA mice by MACS (CD11c MicroBeads (Miltenyi Biotec)), in the presence of 50 μg/mL of collagen (MD Bioproducts, Zürich, Switzerland) or MBT (Difco) protein. For CD3/CD28-stimulated T cell cultures, CD4^+^CD25^–^ naïve T cells were isolated from naïve DBA1 mice and cultured in the presence of coated anti-CD3 (clone 145-2C11) and soluble anti-CD28 (clone 37.51) antibodies (2.0 and 0.5 μg/mL, respectively). After adding sorted Treg and after 4 days of culture, T cell proliferation was assessed by BrdU incorporation and counting.

Conventional DC (cDC (CD11c^+^)), plasmacytoid DC (mPDCA^+^B220^+^) and macrophages (CD11b^+^) were analyzed in the spleen for ex vivo expression of costimulatory molecules CD40 (clone 3/23, Biolegend) and major histocompatibility class (MHC) II IA/IE (clone M5/114.15.2, Biolegend) molecules by FACS (with the corresponding labeled antibodies). In addition, cDC, pDC and macrophages were isolated by MACS and cultured in the presence of CpG-ODN2216 (for pDC, 12 μg/mL (Invivogen, Toulouse, France)) or lipopolysaccharide (LPS) (for cDC and macrophages, 1 μg/mL (Sigma Aldrich, St Louis, MO, USA)) for 24 h in complete medium and the same marker expression was assessed by FACS. Isolated APC were also cultured with allogeneic naïve CD4^+^CD25^–^ T cells (at a ratio of one APC to two T cells) for 5 to 7 days in complete medium and Foxp3 expression was evaluated by FACS in CD4^+^ T cells according to manufacturer’s instructions.

### Assessment of circulating anti-collagen antibody titers

Concentrations of anti-bovine type II collagen antibodies (IgG2a isotypes) were determined in plasma by enzyme-linked immunosorbent assay (ELISA) [21]. Briefly, 96-well plates were coated with 0.5 μg/mL of bovine type II collagen. Nonspecific binding sites were blocked with a 2 % solution of BSA. Serial dilutions of mouse sera were added followed by incubation with IgG2a-specific goat anti-mouse antibody (horseradish peroxidase (HRP)-labeled) and 3,3'-5,5' tetramethylbenzidin (TMB) (MOSS Inc., Pasadena, MD, USA) as substrate. Absorbance was measured at 450 nm.

### Histological analysis

Whole hind legs were harvested, cut through the sagittal axis, fixed with 10 % neutral formalin, decalcified using rapid decalcifier solution (Eurobio, Les Ulis, France), and embedded in paraffin. Serial tissue sections (5 μm) were stained with hematoxylin, eosin and saffron (Merck, Darmstadt, Germany). Joint inflammation was evaluated blinded by a research pathologist and determined in the lower ankle of the two hind legs with a cumulative score at high magnification, looking at cartilage (0 = no change, 1 = erosion, 2 = absence) and synovial inflammation (0 = no infiltrate, 1 = middle mononuclear cell infiltrate, 2 = dense mononuclear cell infiltrate), and a global score for the ankle at low magnification from 0 to 5, with a maximum score of 18 per mouse.

### Statistical analysis

All data were analyzed with Prism 5 (GraphPad; La Jolla, CA, USA) or SigmaStat 3.5 (Systat Software Inc; London, UK) software. Statistical significance was determined by the indicated adequate tests.

## Results

### Treatment of arthritic mice with intravenous apoptotic cell infusion reduces arthritis clinical score

**Fig. 1:**
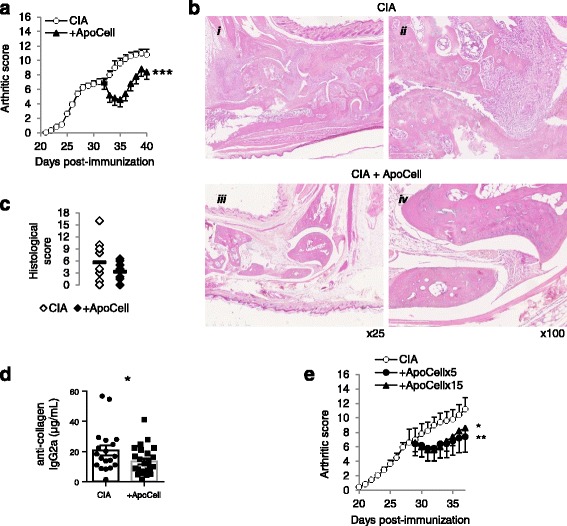
Apoptotic cell injection modulates the severity of collagen-induced arthritis (*CIA*). CIA mice received 5.10e6 apoptotic cells (ApoCell), or 15.10e6, or vehicle (CIA), when they demonstrated an arthritis score of 7–8 and were monitored daily (**a**, **e**). Data are expressed as mean of group ± SEM from 5 to 10 individual animal from one to two independent experiments; *p < 0.05, **p < 0.01 and ***p < 0.001 (two-way analysis of variance with Sidak’s multiple comparisons test). At the time the mice were killed, joint cuts were stained with HES, observed at × 25 and × 100 magnification (**b**), histological lesions were scored in the rear ankles (**c**) (8 to 9 mice from two independent experiments; no difference, Mann-Whitney test), and anti-collagenase IgG2a antibodies were quantified in the serum (**d**) (19 to 25 mice per group from four to five independent experiments; *p < 0.05, parametric paired *t* test)

We have previously shown that apoptotic cell injection allows the prevention of streptococcal cell-wall-induced arthritis in rats [11]. This time we evaluated whether apoptotic cell injection would allow the control of ongoing arthritis. Therefore, CIA mice with a moderate to intense clinical score (7–8 out of 16) received an apoptotic cell or vehicle i.v. injection. Soon after injection, the clinical score in the apoptotic cell-treated CIA mice was strongly reduced compared to mice receiving vehicle (Fig. 1a). During the next week, the clinical score remained low and did not reach the clinical score level of control CIA mice (Fig. 1a). Indeed, when the mice were killed we observed that the severity of histological lesions of the joints tended to decrease, notably with hyperplasia of the synovia and cartilage erosion (Fig. 1b, c). In addition, we observed that the levels of anti-collagen IgG2a antibodies were reduced in CIA mice treated by apoptotic cells (Fig. 1d). Because apoptotic cell injection allowed the control of the progression of arthritis, we then evaluated whether injecting a higher number of apoptotic cells would increase this effect. Again, CIA mice with a score of 7–8 received 5 or 15.10e6 apoptotic cells, or vehicle, and the arthritis clinical score was evaluated daily. We did not observe any additional benefit in the reduction of the arthritis clinical score when more apoptotic cells were infused (Fig. 1e). Indeed, the injection of three times more apoptotic cells did not further decrease the severity of arthritis (Fig. 1e). This confirms our previous data showing that apoptotic cell injection can alleviate arthritis [11] and reinforces the fact that 5.10e6 apoptotic cell infusion is sufficient to affect the inflammatory response [11, 17, 20, 22].

### Intravenous apoptotic cell injection modifies T cell response to collagen auto-antigen

**Fig. 2:**
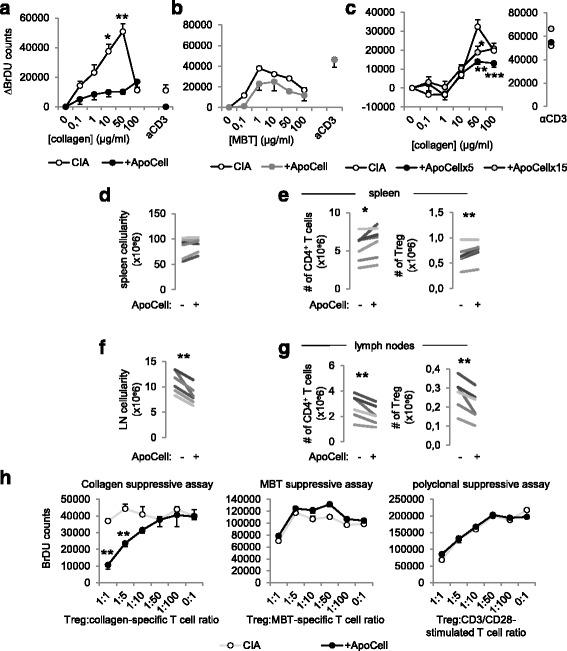
Apoptotic cell injection favors the induction of collagen-specific T regulatory cells (*Treg*). Cells from the lymph nodes of collagen-induced arthritis mice (*CIA*) receiving or not receiving apoptotic cell injection (*+ApoCell*) were harvested at sacrifice and cell proliferation was assessed through 5-Bromo-2’-deoxyuridine (*BrDU*) incorporation and counting in the presence of increasing concentrations of collagen antigen, *Mycobacterium tuberculosis* (*MBT*), or CD3-specific antibodies, as indicated (**a**-**c**). Data are shown as mean ± SEM from one representative experiment out of three with five mice per group; *p < 0.05, **p < 0.01, ***p < 0.001, vs. CIA group (nonparametric unpaired *t* test). In addition, cellularity was assessed, and CD4^+^ T cell and Foxp3^+^ Treg percentages and absolute numbers in the spleen (**d**, **e**) and lymph nodes (*LN*) (**f**, **g**) were determined. Data are shown as means from seven independent experiments; each experiment includes five mice per group; *p < 0.05, **p < 0.01 (parametric paired Student *t* test). Suppressive assays were performed using Treg isolated from the spleens of the previous treated and untreated CIA mice, and added at a different ratio into collagen-specific or MBT-specific co-cultures, or in CD3/CD28-stimulated T cell cultures, and responder T cell proliferation was assessed by BrdU incorporation and counting (**h**). Data are shown as mean ± SEM from one experiment with five mice per group; **p < 0.01, nonparametric unpaired *t* test

At days 8 to 10 post treatment, spleen cells were collected and cultured for 4–5 days with increasing concentrations of collagen protein to evaluate collagen-specific T cell proliferative response. We observed that cell proliferation in response to collagen stimulation was strongly inhibited with cells from apoptotic cell-treated mice compared to cells from untreated arthritic mice (Fig. 2a). This difference was not observable using a polyclonal stimulus, CD3-specific antibody (Fig. 2a). More importantly, we took advantage of the MBT antigen, mixed with collagen in CFA for induction of arthritis, to analyze T cell response against another antigen, and we observed similar cell proliferation against MTB antigen between cells from apoptotic cell-treated and untreated CIA mice (Fig. 2b). This strongly confirms that reduced T cell proliferation associated with apoptotic cell injection was related to a collagen-specific immunomodulation. Collagen-specific immunomodulation was also observed with the cells from CIA mice receiving 15.10e6 apoptotic cells (Fig. 2c).

The collagen-specific immunomodulation that we observed in association with the injection of apoptotic cells suggests the induction of collagen-specific Treg, therefore limiting antigen-specific T cell proliferation in response to collagen. Although we observed similar numbers of mononuclear cells in the spleen in CIA mice receiving or not receiving apoptotic cells (Fig. 2d), we noted that the absolute numbers of CD4^+^ T cells and Foxp3^+^CD25^+^CD4^+^ Treg were increased in the spleen in CIA mice receiving apoptotic cells (Fig. 2e). In the lymph nodes in contrast, cellularity (Fig. 2f) and the absolute numbers of CD4^+^ T cells and Treg (Fig. 2g) were decreased in CIA mice receiving apoptotic cells. The role of Treg in collagen-specific immunomodulation was further investigated using sorted Treg, added into collagen-specific or MBT-specific co-cultures or CD3/CD28-stimulated T cell culture, at a different ratio. Our data showed that only Treg sorted from apoptotic cell-treated CIA mice allowed the suppression of the collagen-specific T cell response, but not the Treg sorted from untreated CIA mice (Fig. 2h, left panel). In addition, the suppressive activity of the Treg sorted from apoptotic-cell-treated CIA mice was restricted to collagen antigen and not extended to MBT antigen, as Treg from CIA or apoptotic-cell-treated CIA mice demonstrated the same ability to limit both MBT-specific co-culture and CD3/CD28-stimulated T cell culture proliferation (Fig. 2h, middle and right panels). Our data strongly demonstrate that the infusion of apoptotic cells allows the induction of Treg in vivo with an antigenic specificity restricted to the collagen autoantigen.

### Apoptotic cell injection modulates the activation of antigen presenting cells

**Fig. 3:**
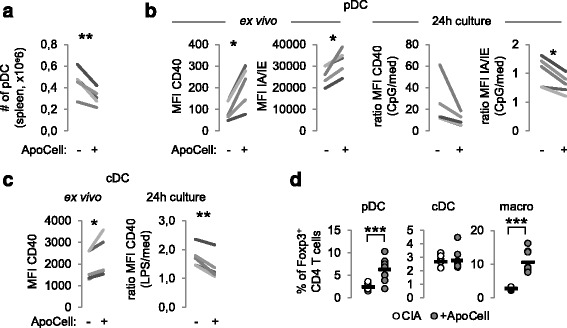
Apoptotic cell injection favors pro-regulatory properties to antigen presenting cells (APC). Plasmacytoid dendritic cells (*pDC*), conventional DC (*cDC*) and macrophages (*macro*) were isolated from the spleen of collagen-induced arthritis (*CIA*) mice receiving or not receiving apoptotic cells (*+ApoCell*) and were quantified (**a**), evaluated for costimulatory and class II molecules (*CD40*, *IAIE*, respectively) expression ex vivo or after stimulation by adequate toll-like receptor ligand (**b**, **c**), and cultured with naïve allogeneic CD4^+^ T cells to evaluate T regulatory cell (Treg) induction (**d**). Data are shown as means from seven independent experiments; each experiment includes five mice per group; *p < 0.05, **p < 0.01 (parametric paired Student *t* test) (**a**-**c**). **d** Data from individual mouse performed in triplicate plus mean (*black bar*) from one of three representative experiments; ***p < 0.001 (parametric unpaired *t* test). *MFI* mean fluorescence intensity

The immunomodulatory properties of apoptotic cells, and notably the induction of Treg, have been associated with phagocyte reprogramming [7, 17, 23]. Indeed, apoptotic cell efferocytosis generates an anti-inflammatory microenvironment containing TGF-β [23, 24]. Plasmacytoïd dendritic cells have been also shown to play a major role in apoptotic cell-induced immunomodulation [20]. We therefore analyzed pDC, cDC and macrophages 10 days after apoptotic cell injection in CIA mice and we observed that the number of pDC was significantly decreased in the spleen of apoptotic cell-treated CIA mice (Fig. 3a). Although pDC from apoptotic cell-treated CIA mice expressed more CD40 and class II IA/IE molecules ex vivo, they responded less to CpG stimulation during a 24-h culture period (Fig. 3b). Concerning cDC, we also observed differential expression of CD40 costimulatory molecules ex vivo when cDC were issued from apoptotic cell-treated mice (Fig. 3c). They were also less sensitive to LPS-induced activation when from apoptotic cell-injected CIA mice (Fig. 3c). For macrophages there was no difference in costimulatory molecule expression and response to LPS-induced activation (data not shown). Altogether, the data show that despite the ongoing arthritic inflammatory response, apoptotic cell injection modifies pDC and cDC functions in vivo, which then respond less to TLR ligands ex vivo.

We further evaluated APC reprograming, i.e., whether APC modulation by apoptotic cells in vivo was imprinted in the APC. This was addressed by culturing sorted APC with naïve CD4^+^ T cells, and then evaluating Foxp3 acquisition in CD4^+^ T cells by FACS analysis. We observed that when cultured with pDC or macrophages isolated from CIA mice receiving apoptotic cells, naïve CD4^+^ T cells demonstrated a strong commitment to the Treg profile as attested by Foxp3 expression (Fig. 3d), but not with cDC from apoptotic-cell-treated CIA mice (Fig. 3d, middle panel). The data demonstrate that apoptotic cell injection modulates in-depth APC behavior in vivo with acquisition of pro-regulatory properties, or at least pDC and macrophages undergo reprogramming.

### Apoptotic cell injection-induced modulation of arthritis is dependent on transforming growth factor-β

**Fig. 4:**
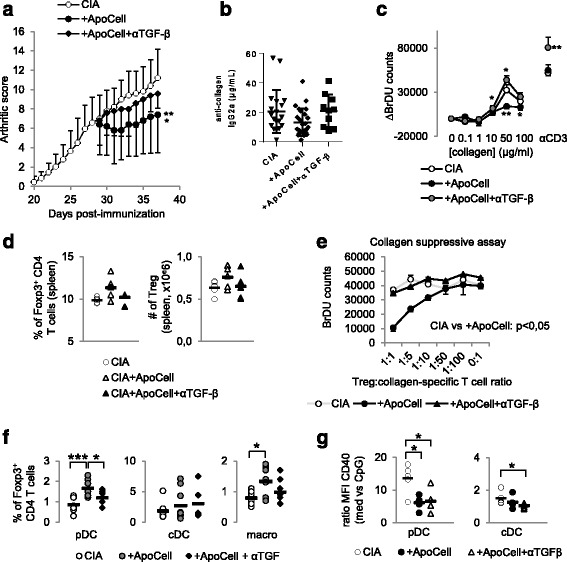
Apoptotic cell-induced immunomodulation in mice with collagen-induced arthritis (*CIA*) is dependent on transforming growth factor (*TGF*)-β. CIA mice that received or did not receive apoptotic cells, with or without anti-TGF-β blocking antibody were scored daily (**a**). Data are shown as mean ± SEM of five mice per group from one of two representative experiments; *p < 0.05, **p < 0.01 (Friedman test analysis of variance (ANOVA) with Dunn's multiple comparisons test). Anti-collagen IgG2a antibodies (Ab) were quantified in plasma (**b**). Data from 5 to 16 mice from two to three independent experiments (no difference, Kruskal-Wallis test ANOVA with Dunn’s multiple comparisons test). Lymph node cells were collected, cultured, and T cell proliferation in response to collagen protein or CD3-specific Ab stimulations was evaluated (**c**). Data are shown as mean ± SEM from one of two representative experiments; *p < 0.05, **p < 0.01 (nonparametric unpaired *t* test). Percent and absolute number of Foxp3^+^ T regulatory cells (*Treg*) were evaluated in the spleen (**d**) and suppressive assays were performed by isolating and adding Treg at a different ratio into collagen-specific cultures and responder T cell proliferation was assessed (**e**). Data are shown as mean ± SEM from one experiment (**d** Kruskal-Wallis test ANOVA with Dunn’s multiple comparisons test; **e** Friedman test ANOVA with Dunn’s multiple comparisons test). Plasmacytoid dendritic cells (*pDC*), conventional dendritic cells (*cDC*) and macrophages (*macro*) were isolated and cultured with naïve allogenic T cells to determine Treg polarization (**f**), and their response to TLR ligand was assessed through evaluation of CD40 costimulatory molecule expression (**g**). Data from individual mouse plus mean (*black bar*) from one experiment representative of four are shown; *p < 0.05, ***p < 0.001 (**f** ordinary one-way ANOVA with Tukey’s multiple comparisons test; **g** Kruskal-Wallis test ANOVA with Dunn’s multiple comparisons test). *BrDU* 5-Bromo-2’-deoxyuridine

TGF-β is a master anti-inflammatory cytokine involved in many processes to maintain immune tolerance and is associated with the direct and indirect immunomodulatory properties of apoptotic cells. Thus, on the day of apoptotic cell injection, mice also received neutralizing anti-TGF-β antibody or control isotype antibody. Although CIA mice receiving apoptotic cells plus isotype demonstrated a reduction and stabilization of the disease, CIA mice receiving apoptotic cells plus anti-TGF-β antibody demonstrated no reduction but an arthritic evolution similar to control CIA mice (Fig. 4a). As a consequence, levels of anti-collagen IgG2a antibodies were observed similar to those observed in control untreated CIA mice when TGF-β was neutralized in apoptotic cell-treated CIA mice (Fig. 4b). This demonstrates that apoptotic cell-induced immunomodulation is associated with TGF-β.

As we observed association between alleviation of apoptotic-cell-induced arthritis and Treg induction, we then looked at collagen-specific cell proliferation and observed that TGF-β neutralization restored apoptotic-cell-induced limited proliferation to collagen (Fig. 4c). These data suggest that TGF-β neutralization inhibits apoptotic cell-induced collagen-specific Treg; we then looked at Treg in the spleen and observed similar percentages and absolute numbers of Treg in apoptotic cell-treated CIA mice receiving anti-TGF-β antibody compared to untreated CIA mice (Fig. 4d). In addition, when we looked at the antigen specificity of Treg issued from CIA mice treated with apoptotic cells and in the presence of TGF-β neutralization, we did not observe any suppressive effect of such Treg in collagen-stimulated cell co-culture (Fig. 4e). This confirms that apoptotic cell-induced collagen-specific Treg in vivo are dependent on TGF-β. To further evaluate whether apoptotic cell-induced Treg inhibition by TGF-β neutralization was associated with blockade of APC reprogramming by apoptotic cells, pDC and macrophages issued from CIA mice receiving apoptotic cells and anti-TGF-β antibody were cultured with naïve CD4^+^ T cells and these were less efficient in favoring Treg commitment (Fig. 4f). However, when we looked at ex vivo APC response to TLR ligands, we observed that in vivo neutralization of TGF-β did not inhibit apoptotic cell-induced cDC or pDC resistance to TLR ligand activation (Fig. 4g). These data demonstrate that TGF-β is implicated in the generation of collagen-specific Treg after apoptotic cell injection and in the acquisition of pro-tolerogenic properties by pDC and macrophages. In addition, our data highlight that apoptotic cell-induced resistance of cDC and pDC to TLR stimulation is independent of TGF-β.

### Methotrexate does not interfere with intravenous apoptotic-cell-based therapy

**Fig. 5:**
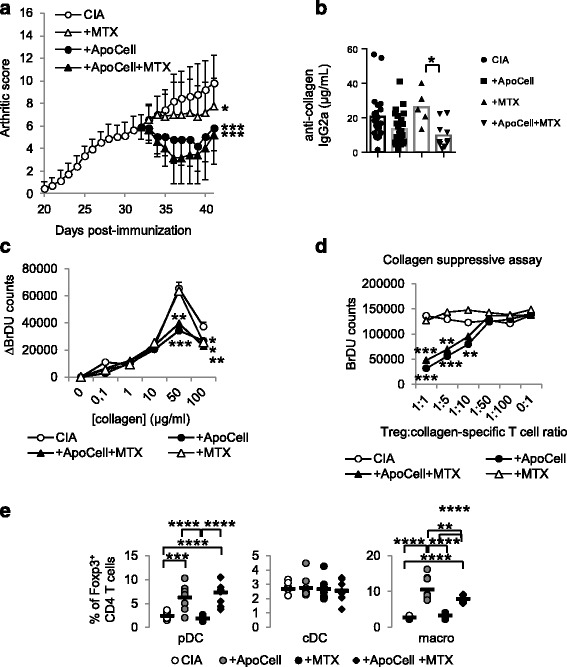
Methotrexate (*MTX*) does not inhibit ​apoptotic cell-induced  reduction of arthritis severity. Collagen-induced arthritis (*CIA*) mice received or did not receive apoptotic cells, with or without MTX, or MTX alone and were scored daily (**a**). Data are shown as mean ± SEM of five mice per group from one of two representative experiments; *p < 0.05 vs. CIA, ***p < 0.001 (two-way analysis of variance (ANOVA) with Sidak's multiple comparisons test). Anti-collagen IgG2a antibodies (Ab) were quantified in plasma (**b**). Data from 5 to 16 mice from two independent experiments plus mean (*bar graph*); *p < 0.05 (Kruskal-Wallis test ANOVA with Dunn's multiple comparisons test). Lymph node cells were collected, cultured and T cell proliferation was evaluated in response to collagen antigen or CD3-specific Ab stimulations (**c**). Data are shown as mean ± SEM from one of two representative experiments; *p < 0.05, **p < 0.01 (nonparametric unpaired *t* test). T regulatory cells (*Treg*) were isolated and added at different ratio into co-cultures specific for collagen and responder T cell proliferation was assessed (**d**). Data are shown as mean ± SEM from one experiment; **p < 0.05, ***p < 0.001 (nonparametric unpaired *t* test). Plasmacytoid DC, cDC and macrophages were isolated and cultured with naïve allogeneic T cells to determine Treg polarization (**e**). Data from individual mouse plus mean (*black bar*) from one experiment representative of two are shown; **p < 0.01, ***p < 0.001, ****p < 0.0001 (ordinary one-way ANOVA with Tukey’s multiple comparisons test). *BrDU* 5-Bromo-2’-deoxyuridine

To propose apoptotic cell injection in humans as an advanced therapeutic medicinal product to alleviate RA, we then evaluated apoptotic cell therapy with conventional RA treatment, MTX. Arthritic mice receiving MTX treatment alone had slight reduction in disease (*p* < 0.05 vs. CIA) compared to CIA mice receiving apoptotic cell injection, which strongly decreased the arthritis score (*p* < 0.001 vs. CIA) (Fig. 5a). Co-treatment, apoptotic cells plus MTX, demonstrated disease reduction similar to apoptotic cell injection alone (Fig. 5a), suggesting that MTX does not interfere with ​apoptotic cell-induced reduction of arthritis. We also observed that co-treatment reduced collagen-specific IgG2a antibody levels (Fig. 5b). Looking at collagen-specific T cell proliferation, co-treatment also allowed collagen-specific immunomodulation (Fig. 5c). This demonstrates that MTX does not inhibit apoptotic cell-induced collagen-specific immunomodulation. Whereas spleen CD4^+^ T cell and Treg numbers were not affected by MTX/apoptotic cell co-treatment (not shown), we observed that Treg issued from co-treated CIA mice also demonstrated collagen-specific suppressive functions (Fig. 5d), confirming that MTX does not inhibit apoptotic cell-induced collagen-specific Treg induction. In addition, when we assessed APC reprogramming, we observed that MTX/apoptotic cell co-treatment did not inhibit apoptotic cell-induced APC reprogramming in vivo, as pDC and macrophages from co-treated CIA mice demonstrated ex vivo properties to favor Treg commitment from naïve CD4^+^ T cells (Fig. 5e). These data demonstrate that apoptotic cell co-treatment with MTX further decreases the severity of arthritis compared to MTX alone, and that MTX does not affect apoptotic cell-induced collagen-specific Treg induction and APC reprogramming mechanisms.

### Apoptotic cell-based therapy synergizes with anti-TNF antibody in collagen-induced arthritis in mice

**Fig. 6:**
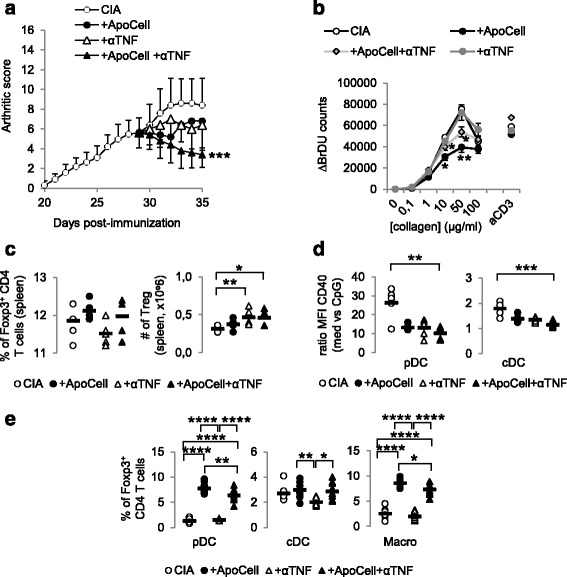
Apoptotic cell injection and anti-TNF therapy synergize to control ongoing arthritis. Collagen-induced arthritis (*CIA*) mice received or did not receive apoptotic cells, with or without anti-TNF blocking antibody and were scored daily (**a**). Data are shown as mean ± SEM of five mice per group from one experiment; *p < 0.05 (two-way analysis of variance (ANOVA) with Sidak's multiple comparisons test). Lymph node cells were collected, cultured, and T cell proliferation was evaluated in response to collagen protein or CD3-specific antibody (Ab) stimulations (**b**). Data are shown as mean ± SEM from one experiment; *p < 0.05, **p < 0.01 vs. CIA (nonparametric unpaired *t* test). Percentages and absolute numbers of Foxp3 T regulatory cells (*Treg*) were evaluated in the spleen (**c**). Data are shown as individual mouse plus mean (*black bar*) from one experiment; *p < 0.05, **p < 0.01 (Kruskal-Wallis ANOVA test with Dunn’s multiple comparisons test). Plasmacytoid dendritic cells (*pDC*), conventional dendritic cells (*cDC*) and macrophages (*Macro*) were isolated to evaluate their response to toll-like receptor (TLR) ligand stimulation through evaluation of CD40 costimulatory molecule expression (**d**) and cultured with naïve allogeneic CD4^+^ T cells to determine Treg polarization (**e**). Data from individual mouse plus mean (*black bar*) from one experiment; *p < 0.05, **p < 0.01, ***p < 0.001, ****p < 0.0001(**d** Kruskal-Wallis ANOVA test with Dunn’s multiple comparisons test, **e** ordinary one-way ANOVA test with Tukey’s multiple comparisons test) *BrDU* 5-Bromo-2’-deoxyuridine, *MFI* mean fluorescence intensity

To further address the feasibility of apoptotic cell injection in RA, we evaluated apoptotic cell injection and anti-TNF antibody effect. Apoptotic cell injection or anti-TNF blockade demonstrated a similar reduction in arthritis (Fig. 6a), but, when co-administered, apoptotic cells and anti-TNF antibody demonstrated a stronger reduction in the severity of arthritis (Fig. 6a). Looking at the mechanisms, whereas anti-TNF antibody treatment alone did not favor collagen-specific immunomodulation compared to apoptotic cell injection, apoptotic cell/anti-TNF antibody co-injection induced collagen-specific immunomodulation, but to a lower extent to that observed with apoptotic cell injection alone (Fig. 6b). Also, anti-TNF antibody treatment favored a higher number of spleen Treg, that were maintained in the presence of apoptotic cells (Fig. 6c). So far, our data demonstrate that apoptotic cell injection with TNF neutralization strongly reduces arthritis severity and allows an increased number of spleen Treg.

When APC reprogramming was evaluated, pDC and cDC demonstrated less response to TLR ligand-induced activation when issued from CIA mice receiving anti-TNF antibody, with or without apoptotic cell injection (Fig. 6d). Macrophage response to TLR was again not affected by either treatment (not shown). When issued from anti-TNF/apoptotic cell-treated CIA mice, pDC and macrophages then allowed Treg commitment from naïve CD4^+^ T cells (Fig. 6e). This was not so with anti-TNF antibody injection alone (Fig. 6e). Our data demonstrate that anti-TNF antibody treatment alone modulates arthritis severity by favoring increase in Treg but not collagen-specific Treg or APC reprogramming with pro-regulatory properties. When administered with apoptotic cells, a stronger modulation of arthritis severity was observed associated with a strong reduction of collagen-specific T cell response and the gain of pro-regulatory properties by APC.

## Discussion

Despite significant improvement in the management of patients with RA through the development of innovative drugs (e.g., biologic agents) [25, 26], new therapeutic strategies are constantly needed. Indeed, some patients are still non-responsive to the current drugs and toxicities, or contraindications have been reported [4, 26]. Most of the biologic agents target inflammatory cytokines or B cells, while limited biologic agents (i.e., abatacept) neutralize T cell activation [25, 26]. There is no approved drug that targets APC, such as dendritic cells, while APC orchestrate the immuno-pathogenic response.

Apoptotic cell-based therapy can be an interesting approach as apoptotic cell infusion has been shown to prevent experimental RA [9–12] and to target T-cell-mediated diseases associated with dysregulated inflammatory cytokine secretion and APC dysfunction [6]. A dysregulated intricate interplay between T cells, macrophages and inflammatory cytokines is implicated in RA pathophysiology. Here, we used a mouse model of arthritis that recapitulates RA [27]. We report in this model that i.v. administration of apoptotic cells is able to treat ongoing arthritis and is associated with the reprogramming of APC, in particular pDC and macrophages, and induction of auto-antigen-specific Treg. Moreover, this new therapeutic approach can be used alongside either MTX or anti-TNF therapy (i.e., two standard treatments of RA) with similar efficacy. This allows us to propose this approach in clinical settings, as already performed for the prevention of GvHD after allogeneic hematopoietic cell transplantation [16]. As observed in mice, patients would have to receive such a treatment when presenting with a moderate 20 % improvement in the American College of Rheumatology (ACR20) clinical score for a higher therapeutic effect.

The novelty of this study resides in the use of apoptotic cell infusion to treat ongoing arthritis, and not, as previously reported by us [11] and others [9, 10, 12], to prevent the induction of arthritic disease. The analysis of the mechanisms involved in this therapeutic effect of i.v. apoptotic cell infusion identifies the modulation of APC functions and reprogramming, induction of auto-antigen-specific Treg, and reduction of circulating auto-antibody levels. Adoptive transfer of polyclonal Treg has been already shown to inhibit ongoing arthritis in the CIA model, but this approach only ameliorated the severity of CIA without affecting collagen-specific T and B cell responses [28]. This is in contrast with our present data showing that apoptotic cell infusion through the direct targeting of APC function affects collagen-specific immune response. The CIA model, and more precisely the immunization of mice with bovine type II collagen in CFA containing MBT, allowed us to appreciate the antigenic specificity of apoptotic cell-induced Treg with a selective induction of collagen-specific Treg, but not MBT-specific Treg. The precise mechanisms allowing this specific increase in auto-antigen Treg (here collagen as the model antigen), but not infectious antigen (MBT), remains to be determined. One may believe that distinct APC subsets or the antigen-presenting pathways are in charge of auto-antigen or infectious antigen and that this subset or pathway is differentially targeted by apoptotic cell infusion. This remains to be determined.

We previously observed that pDC were sensitive to factors issued from macrophages eliminating apoptotic cells but not to apoptotic cells directly [20], in contrast to DC or macrophages [23]. Here, macrophages will be the first ones to interact and eliminate apoptotic cells injected i.v., in contrast to cDC. Then, as previously reported [20], this microenvironment provided by macrophages eliminating apoptotic cells will directly favor pDC to acquire a tolerogenic profile, notably with the *de novo* production of TGF-β, favoring pDC to allow generation of antigen-specific Treg [19]. However, this microenvironment only allowed cDC to favor less Th1 polarization ex vivo (not shown). It seems that apoptotic cells differentially affect cDC, as when engulfed by cDC they favored cDC pro-tolerogenic reprogramming, but when engulfed by macrophages, the issued factors only limited their pro-inflammatory properties.

Similar observations have been performed in another apoptotic cell-based therapy implicating in vivo induction of apoptosis and phagocyte administration together with auto-antigen-derived peptides [29]. Despite differences in the process and the targeted diseases (EAE and type 1 diabetes), this latter approach described similar mechanisms to our therapy, including: apoptotic cell phagocytosis, TGF-β-dependent Treg generation [29].

Induction of collagen-specific Treg by apoptotic cell injection is inhibited by TGF-β neutralization, as previously reported in other apoptotic cell-based therapies [17, 29]. Most of the studied effects observed here after apoptotic cell treatment are dependent on TGF-β and neutralization of this cytokine prevents improvement in arthritis. Anti-collagen IgG2a antibody levels reached the levels found in untreated arthritic mice when neutralizing anti-TGF-β antibody is infused together with apoptotic cells. This confirms our previous data with anti-donor allo-antibodies in the settings of bone marrow graft rejection [30]. In fact, only APC reprogramming persisted after neutralization of TGF-β in the setting of apoptotic cell infusion in arthritic mice. This appears logical as TGF-β is secreted by APC after apoptotic cell removal [23, 31] but elimination of apoptotic cells (called efferocytosis) per se is sufficient to modify several APC functions, including the refractory response to TLR ligands [32]. The effects on APC functions induced after apoptotic cell treatment are conserved when apoptotic cells are infused together with anti-TNF therapy. This can be an encouraging argument to combine both treatments.

Association of new therapeutic approaches with drugs used routinely is infrequently assessed in experimental models. To the best of our knowledge, this has only been tested in the context of apoptotic cell infusion in one study by our group in the settings of allogeneic hematopoietic cell transplantation [18]. Here, we reported that the therapeutic effects of apoptotic cell infusion are conserved in the presence of MTX or anti-TNF therapy. However, there are differences in apoptotic cell-induced mechanisms. For instance, MTX abolishes the diminution of anti-collagen auto-antibodies induced by apoptotic cell injection. MTX, a traditional DMARD with folate antagonist and anti-inflammatory activity, remains the gold standard treatment for RA. Several potential mechanisms of MTX activity have been proposed, but the precise mechanism(s) of action in RA are still not well-understood [33]. In animal models, the effects of MTX have been attributed to MTX-induced anti-inflammatory adenosine production [33]. This production is mediated by CD39 and CD73 ectoenzymes [33]. Both ecto-enzymes are known to be expressed by Treg and represent one of the suppressive mechanisms of Treg [34].

MTX treatment demonstrated a limited effect on arthritis severity when started in mice presenting with an advanced arthritic clinical score of 8. In this context, after the injection of apoptotic cells, collagen-specific Treg are still generated. We can propose that apoptotic cell therapy in CIA favors the generation of Treg from existing antigen-specific T cells, as no cell proliferation is needed and MTX can exert an anti-proliferative effect even if the cytostatic effect of MTX is still a matter of debate in RA [33]. However, the origin of antigen-specific Treg in our settings needs to be further investigated. Thus, through the induction of an anti-inflammatory microenvironment, apoptotic cells can favor the establishment of collagen-specific Treg-associated long-term tolerance. Anti-TNF therapy is synergic with apoptotic cell infusion on clinical outcomes, but we did not identify any supplementary effects on the immune parameters studied when anti-TNF antibody is combined with apoptotic cells compared with apoptotic cells alone. This suggests that additional mechanism(s) such as the complete neutralization of circulating TNF, may participate in the synergic clinical outcomes. The relationship between TNF, anti-TNF therapy and Treg plasticity and suppressive functions is complex and remains a matter of debate [35], notably in humans [36, 37]. Here, anti-TNF therapy does not affect the number of splenic Treg or the collagen-specific Treg suppressive function.

## Conclusions

We provide here a novel medicinal product for advanced therapy for RA, which is compatible with first-line MTX treatment and synergistic with anti-TNF biological second-line treatment. Through the generation of antigen-specific Treg by APC reprogramming by apoptotic cell efferocytosis, this approach offers an innovative therapeutic solution to patients with RA that is refractory and non-responsive to current treatments.

## Abbreviations

7-AAD, 7-aminoactinomycin D2; Ab, antibodies; APC, antigen presenting cells; BrdU, 5-Bromo-2’-deoxyuridine; BSA, bovine serum albumin; CFA, complete Freund adjuvant; CIA, collagen-induced arthritis; cDC, conventional dendritic cells; DC, dendritic cells; DMARD, disease-modifying anti-rheumatic drug; DMEM, Dulbecco’s modified Eagle’s medium; FACS, fluorescence-activated cell sorting; FCS, fetal calf serum; GvHD, graft-versus-host disease; IFN, interferon; i.p., intraperitoneally; i.v., intravenous; LPS, lipopolysaccharide; MBT, mycobacterium tuberculosis; MTX, methotrexate; PBS, phosphate-buffered saline; pDC, plasmacytoid dendritic cells; RA, Rheumatoid Arthritis; TGF-β, transforming growth factor-β; TLR, toll-like receptor; TNF, tumor necrosis factor; Treg, regulatory T cells
